# A Mixed-Method Study Exploring Experiences and Perceptions of Nutritionists Regarding Use of an Image-Based Dietary Assessment System in Tanzania

**DOI:** 10.3390/nu14030417

**Published:** 2022-01-18

**Authors:** Naomi Saronga, Idda H. Mosha, Samantha J. Stewart, Saidah Bakar, Bruno F. Sunguya, Tracy L. Burrows, Germana H. Leyna, Marc T. P. Adam, Clare E. Collins, Megan E. Rollo

**Affiliations:** 1 Priority Research Centre in Physical Activity and Nutrition, The University of Newcastle, Callaghan, NSW 2308, Australia; Naomi.Saronga@uon.edu.au (N.S.); Sam.Stewart@newcastle.edu.au (S.J.S.); tracy.burrows@newcastle.edu.au (T.L.B.); marc.adam@newcastle.edu.au (M.T.P.A.); clare.collins@newcastle.edu.au (C.E.C.); 2 School of Health Sciences, College of Health, Medicine & Wellbeing, The University of Newcastle, Callaghan, NSW 2308, Australia; 3 Department of Community Health, Muhimbili University of Health and Allied Sciences, Dar es Salaam P. O. Box 65015, Tanzania; saidahmohamed2014@gmail.com (S.B.); sunguya@gmail.com (B.F.S.); 4 Department of Behaviour Sciences, Muhimbili University of Health and Allied Sciences, Dar es Salaam P. O. Box 65015, Tanzania; ihmosha@yahoo.co.uk; 5 Hunter Medical Research Institute, Lot 1 Kookaburra Circuit, New Lambton Heights, NSW 2305, Australia; 6 Tanzania Food and Nutrition Centre, Dar es Salaam P.O. Box 977, Tanzania; gerryleyna@yahoo.com; 7 Department of Epidemiology & Biostatistics, Muhimbili University of Health and Allied Sciences, Dar es Salaam P. O. Box 65015, Tanzania; 8 School of Information and Physical Sciences, College of Engineering, Science and Environment, The University of Newcastle, Callaghan, NSW 2308, Australia

**Keywords:** image-based food record, dietary assessment, low and lower middle-income countries, dietary intake, technology

## Abstract

Due to global advances in technology, image-based food record methods have emerged as an alternative to traditional assessment methods. The use of image-based food records in low and lower-middle income countries such as Tanzania is limited, with countries still using traditional methods. The current study aimed to determine the feasibility of using a new voice and image-based dietary assessment system (VISIDA) in Dar es Salaam, Tanzania. This mixed-method study recruited 18 nutritionists as participants who collected image-based records of food and drinks they consumed using the VISIDA smartphone app. Participants viewed an online demonstration of the VISIDA web platform and the analysis process for intake data collected using the VISIDA app. Then, participants completed an online survey and were interviewed about the VISIDA app and web platform for food and nutrient intake analysis. The method was reported as being acceptable and was found to be easy to use, although technical challenges were experienced by some participants. Most participants indicated a willingness to use the VISIDA app again for one week or longer and were interested in using the VISIDA system in their current role. Participants acknowledged that the VISIDA web platform would simplify some aspects of their current job. Image-based food records could potentially be used in Tanzania to improve the assessment of dietary intake by nutritionists in urban areas. Participants recommended adding sound-on notifications, using the VISIDA app in both Apple and Android phones, enabling installation from the app store, and improving the quality of the fiducial markers.

## 1. Introduction

Diet has a significant connection to an individual’s health, and therefore, accurate assessment of dietary intake is very important to study the association between diet and health-related outcomes. Dietary assessment is the process of collecting information on food and drink consumed over a specified time, with this information being analysed to determine the intake of nutrients and/or food groups [1]. Furthermore, nutrient intakes are often compared with recommendations to determine the nutritional adequacy of the diet. The most common traditional methods of assessing dietary intake are food records, 24-h recalls, food frequency questionnaires (FFQs), and dietary histories [2].

The limitations reported under each traditional method are well-documented [2,3]. For instance, all rely on the information reported by the subjects themselves, and therefore the accuracy of each relies on the motivation and willingness of subjects to report accurate diet information. FFQs, 24-h recall, and diet history rely on the subject’s ability to recall eating frequency, type of food, and often the portion size consumed [4]. Incomplete reporting can occur if a subject fails to remember the details of all foods and beverages consumed in the reference period of time. For example, a 24-h recall requires the subject to remember all types and amounts of food and beverages consumed in the past 24 h, and FFQ requires the subject to recall all the foods and drinks consumed in the past week, month or year [5].

Due to advancements in technology globally, image-based, image-assisted and web-based dietary assessment methods have arisen in an effort to increase the accuracy of self-reported dietary intake [6,7,8,9]. Specifically for image-based methods, subjects can use a separate digital camera or cameras embedded in other devices such as smartphones to capture the image of food to be eaten [10]. Although image-based food records generally still depend on the subject to self-report, an advantage of this method is that food records are marked with time, noting exactly when the food item was about to be consumed [11]. This approach allows researchers or health care providers to assess the client’s diet, eliminating bias due to memory lapse [10], which is still a problem in other web-based methods (24-h recall and FFQ). In addition, image-based food records do not require the subject to estimate the amount of food using portion size reference images as in other web-based dietary assessment methods [9,12]. The task of quantifying can be undertaken manually by a trained analyst, such as a nutritionist or automated partially or fully through the use of computer vision.

Image-based food records are new methods of assessing dietary intake in low and lower-middle income countries (LLMICs) such as Tanzania, as most countries still use traditional methods. To date, only image-assisted methods, where images are used to complement traditional methods, have been used in LLMICs. Images were found to assist participants to recall their food intake during 24-h recall in previous studies conducted in Uganda [13] and Bolivia [14]. The current study aimed at investigating the feasibility of using a new image-based dietary assessment system, called VISIDA, with nutritionists in Dar es Salaam, Tanzania.

## 2. Materials and Methods

### 2.1. Study Design

This was a feasibility study using mixed methods for data collection in order to comprehensively compile the views of nutritionists on the VISIDA dietary intake assessment system. The study was conducted in August and September 2021.

### 2.2. Participants and Setting

The aim was to recruit 25 nutritionists working in the Dar es Salaam region of Tanzania. Nutritionists were eligible for inclusion if they met the following criteria: aged ≥18 years; involved in the assessment of dietary intake in their current role; had access to a computer with the ability to connect to the internet; and owned an Android smartphone running an operating system (OS) of version 7.0 or above.

### 2.3. Recruitment

Recruitment of nutritionists occurred remotely. The first author (NS) used the professional network Food and Nutrition Association of Tanzania (FONATA) (of which she is a member) to promote the study to network members. An invitation email was sent to the chairperson of FONATA, who circulated it to members on the researcher’s behalf. As instructed in the invitation email, nutritionists who were interested in participating sent an email to the first author, who then sent an individual email response to the nutritionists who had expressed interest, including the participant information statement and consent form. Participants were asked to read the information statement and send any questions by reply email. Interested nutritionists had one week to consider participation. If no response was received, a follow-up email was sent to those who had expressed interest. Those who agreed to participate were asked to email back the signed consent form.

### 2.4. Data Collection Using the VISIDA System

**Figure 1:**
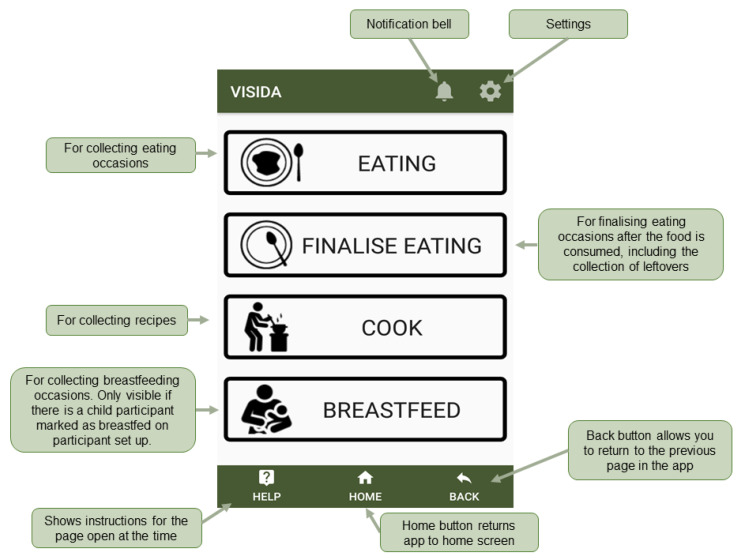
Main features of VISIDA app.

Participants were asked to collect intake data using the VISIDA system, a new image-based dietary assessment system (see https://www.visida.org; accessed on 26 November 2021) designed for LLMIC settings. The system comprises a smartphone app (Figure 1) that allows the trained subject to collect information on their dietary intake in the form of recipes, eating occasions, and leftovers via images and voice recordings. The app contains in-app instructions that remind the subject of the collection process. The collected data from the app is then exported and uploaded to a web-based content management system (VISIDA web platform) for semi-automated analysis of the dietary intake data to produce estimates of nutrient intake using country or region-specific food composition databases. The VISIDA web platform requires an analyst to code the data by identifying and quantifying the uploaded images of food items and recipes. The identification process involves identifying the images in the country or region-specific food composition tables. The quantification process entails assigning measurements on the identified images using either an inbuilt ruler or stored image references with different portion sizes. The platform also allows the comparison of individual nutrient intakes to set recommended dietary intakes.

**Figure 2:**
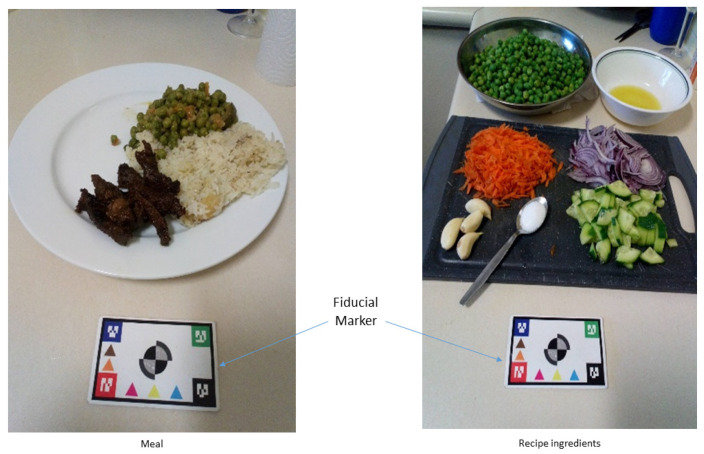
Example image illustrating the collection of food items and ingredients with a fiducial marker.

Participants were instructed to use the app over three days to collect an image of each food and/or drink prior to consumption (Figure 2), along with a voice description of the foods contained in the image. Participants finalised their intake by reporting what happened to the captured food and/or drink and capturing an image and voice recording of any leftovers. A fiducial marker (a reference object with known dimensions, used to assist the quantification of foods included in the images) was placed next to the food items and was included in the picture (Figure 2). The app contains on-screen guidance to centre the fiducial marker and to ensure the image is captured at the optimal angle (~45 degrees). The approach has been previously used in Australia [15,16]. Recipe information on dishes prepared in the home was also captured with the app (Figure 2). Following completion of the intake recording period, participants were asked to export their data from the app and upload it for the study team to access. The activities completed by the participants are summarised in Table 1.

### 2.5. Survey

Following using the app to record their intake, participants completed an online survey. The survey asked questions on (1) sociodemographics such as education level, working experience, job title and groups participants were working with; (2) experience in using a smartphone; (3) experience in using the VISIDA app; (4) capturing photos and voice recordings of all food and drink consumed, as well as recipes, using the VISIDA app; (5) using the app with the population groups participants work with. Sections one and two consisted of multiple-choice questions. For the remaining sections (3–5), participants were requested to select the best option among the 5-point Likert scale (strongly disagree, disagree, neutral, agree, and strongly agree) to respond to the questions.

### 2.6. Interview

After completing the survey, participants were provided with a video demonstration of the VISIDA web platform for analysing dietary intake data collected using the app. The video was sent to them via link by the first author (NS) and was available in both Kiwahili and English languages. The duration taken by each participant to watch the video was monitored. This was to make sure that participants had watched the entire video and therefore standardised the viewing and information provided ahead of the feedback session during the subsequent interview. Participants were interviewed about their experiences in collecting dietary intake data using the app and their perceptions of the analysis process.

Participants were interviewed via video call using the Zoom platform (https://zoom.us accessed on 30 September 2021) to obtain their views on the dietary intake collection app and web platform for nutrient analysis. The interview was guided by four sections: (1) work history information, including profession, responsibilities, working experience, and groups participants were working with; (2) experiences in conducting dietary assessment, including methods and how they use dietary information; (3) experience in using image-based dietary records (VISIDA app), including taking images, recording eating occasions, recording recipes, fiducial marker, notifications, in-app instructions, using the app in their daily work, and challenges; and (4) the VISIDA web platform for analysing nutrient intake data, including the coding process and comparison between intake and recommendations. In total, there were 26 questions. The interview took approximately 45 min and was conducted in Kiswahili.

### 2.7. Analysis

The recorded interviews were transcribed verbatim in Kiswahili and then translated to English. The second author (IHM) supervised the transcriptions and translations. An inductive iterative process was employed by the first and second authors (NS and IHM) to analyse the interview data using four main stages, as identified by Bengtsson [17]: decontextualisation, recontextualisation, categorisation and compilation. Through decontextualization, the transcriptions were read and re-read to gain a sense of its wholeness. After this, data were coded and evaluated for significance. Coding was deductive, using a detailed codebook developed using the study objectives, and inductive, allowing for themes to emerge from the data. Through the process of recontextualisation, the authors re-read the original text together with the final list of codes [17] to make sure all aspects of the content were covered in relation to the aim of the study [17]. Categorization with latent content analysis was used to identify themes. The first author sorted and classified by similar thematic content and separated into smaller categories based on the aims of the study. Lastly, meanings in the text were identified and compiled for presentation. NVivo12 (QSR International Inc., Burlington, MA, USA), a software package for organising, managing, and managing qualitative data, was used to systematically code the interview data.

IBM SPSS statistical software version 27 (IBM Corp., Armonk, NY, USA) was used to derive descriptive statistics and frequencies for data captured in the survey relating to working experience, use of a smartphone, and the experience of using the VISIDA app.

## 3. Results

**Figure 3:**
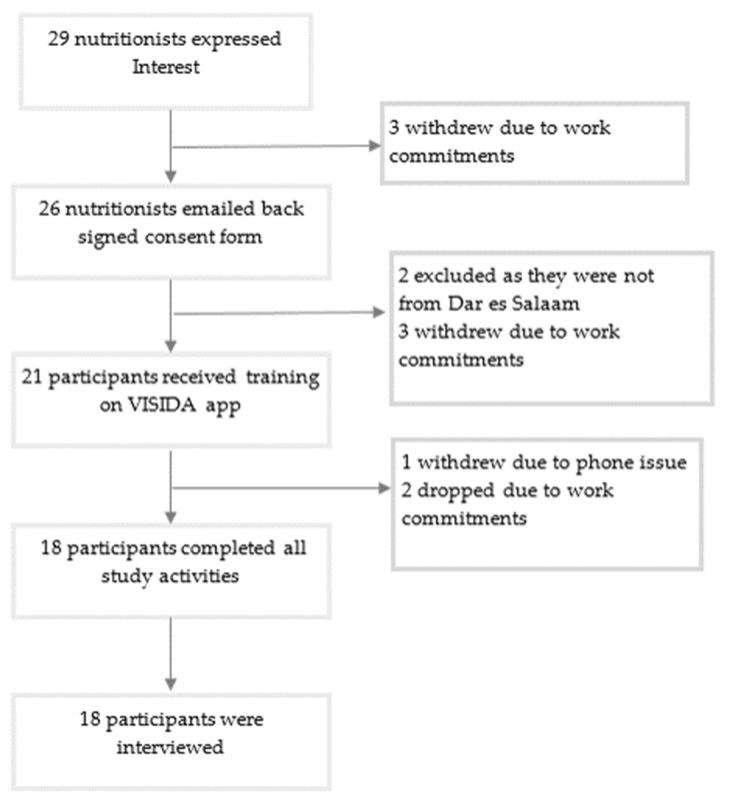
Participant recruitment and flow through the study.

A total of 29 potential participants expressed interest in participating in the study, with 26 consenting to participate and returning the signed consent form. Of this number, 21 participants received training on how to use the VISIDA app to collect their intake. Furthermore, 16 completed an image-based food record for 3 days, and 2 completed a record for 2 days. In total, 18 participants completed all study activities and were included in the analysis. The main reason for participants withdrawing or being lost to follow-up included work commitments/schedule (*n* = 8), not residing in Dar es Salaam (*n* = 2), and a phone issue (*n* = 1). Figure 3 shows the process of recruitment and flow through the study of participants.

### 3.1. Characteristics of the Participants

Participant characteristics are presented in Table 2. Fifteen participants had completed a bachelor’s degree, and three had completed a postgraduate degree. The study recruited 3 males and 15 females. Overall working experience ranged from 1 to 14 years at their current workplace. Ten participants were working in a hospital setting, four were working at a government food and nutrition institution, two were working with a non-governmental organisation, and one was teaching at a secondary school. All participants (*n* = 18) had experience in using a smartphone; 13 (72.2%) reported having a lot of knowledge in using a smartphone. A total of 17 participants (94.4%) reported having experience working with pregnant women, while 11 (61.1%) had experience working with children 13–59 months.

### 3.2. Dietary Assessment Experiences of the Participants

#### 3.2.1. Dietary Assessment and Methods

Participants commonly conducted dietary assessments on their clients using 24-h recalls, food frequency questionnaires, and diet history methods. Participants identified some disadvantages of using these methods, including clients forgetting food items, clients reporting items that they had not eaten, and the difficulty of obtaining accurate information regarding the portion size consumed by their clients.

*“Yes, I do a dietary assessment, but of course, there is a challenge of getting exactly what our client ate. Client can report or mention something different from what she eats or sometimes you find that it [food] doesn’t even exist, s/he is guessing. Sometimes client mentions something thinking that you will help her to get food aid or she will not mention the right things”.*

—P7

*“In many cases, I use 24-h recall and FFQ. Also, dietary history of which we are looking at feeding pattern and the foods that the person eats. Dietary assessment is compulsory. You have to do it”.*

—P13

*“I use 24-h recall……you can’t get correct information, or sometimes you find that a patient has forgotten what she has consumed”.*

—P2

#### 3.2.2. Importance of Assessing Dietary Intake of the Clients

The participants agreed that it is very important to assess dietary intake to identify whether clients meet national recommendations for food group intakes or not in order to plan dietary management. They further stressed that dietary assessment is essential to evaluate whether the client is implementing the dietary advice provided.

*“Dietary assessment helps me to know the reality of the nutrition problems my patients are facing. For example, if it is underweight, dietary assessment methods will help me identifying the dietary challenges that might have caused that nutrition problem”.*

—P3

*“It is essential to assess dietary intake because in that way you will be able to advise the client. You can’t advise the client if you don’t know her dietary intake. You have to understand what the client eats to advise her what to reduce or add in her meals”.*

—P25

#### 3.2.3. Analysis of Dietary Intake Data

Participants acknowledged not analysing dietary intake data because they did not have software to perform the analysis. Instead, participants reported estimating intakes by checking which food groups were/were not consumed by their clients.

*“I don’t have the ability to analyse dietary intake data. I always check whether among these five food groups, which one is not consumed. It can be either patient lacks vegetables or fruits”. *

—P2

*“I look at them, and maybe I used the portion size, I can decide what dominate in clients’ meal. I ask the client to draw on their plate and show me how the plate looks like, and then I anticipate which foods they eat in large amounts and which ones they eat in small quantities; I don’t have software to perform the analysis. In many cases, I do talk to get an idea of what is happening. We don’t do analysis”.*

—P13

### 3.3. Usability of VISIDA App

#### 3.3.1. Data Collection Using VISIDA App

Overall, 13 of 18 participants reported that the VISIDA app was ‘easy to use’; 3 said it was ‘very easy to use’, and 2 were ‘neutral’. All participants said they would like to use the VISIDA app again to collect dietary intake, with eight reporting they would like to use the VISIDA app again for one month and more. Seven participants said they would use the app for up to one week, and the remaining three participants said that they would use it for up to three days. Further responses related to ease of use and level of disruption to daily habits are summarised in Table 3.

From the interview data, all participants concurred that it was easy to record their eating occasions using the VISIDA app. They emphasised that their ease of using the app improved over the recording period. A few participants admitted to forgetting to finalize their eating occasions. The quotes below illustrate these points:

*“To be honest, it was easy recording eating occasions”.*

—P11

*“Recording my voice was easy. I liked that because it was easy”.*

—P10

*“You will try a little bit hard, but sometimes after getting used to it after adapting and being able to balance the fiducial marker and the camera, you can take photos well”.*

—P12

*“I didn’t face any challenge in finalising eating, although I forgot to finalise eating in time until I was reminded”.*

—P10

A few participants managed to record at least one recipe. Those who managed to record indicated that it was easy to record recipes.

*“Recording recipe, to be honest, was good because after preparing my ingredients which are needed, I took photos, then I took voice record to explain which ingredients are in my photos. I completed my cooking and recorded the image of the final dish. It was good”.*

—P4

#### 3.3.2. Notifications

All the participants indicated that notifications were very helpful in reminding them to finalise eating and review the day. However, most participants suggested improving the way notifications appeared by adding an alarm. Some even suggested the notification should appear on their phones as a normal message so that they would not have to go to the app to see the notification message. See the quotes below:

*“Notifications were helpful to me. They reminded me to review my day, and if there was something that I ate and forgot to record, I got a chance to record”.*

—P12

*“Notifications reminded me to finalise eating something which I have frequently been forgetting. Notifications should come directly to the phone to be able to see even if I have not opened the app”.*

—P7

*“The challenge is that they were popping up silently. I think it would be best if I could hear voice or vibrations when the notifications are popping up”.*

—P3

#### 3.3.3. In-App Instructions

Participants agreed that the in-app instructions were helpful, especially when recording their first eating occasion. The instructions reminded them of the steps they had forgotten.

*“In-app instructions are one of the very important sections of this program because human beings tend to forget. It helped me a lot to know where to start and where to end”.*

—P2

*“In-app instructions helped me a lot. They are informative resemble the instructions you sent to me through email on using the VISIDA app. It was like a handout to me. When you have the VISIDA app on your phone, you also have the user operational manual of VISIDA”.*

—P3

#### 3.3.4. Fiducial Marker

Many participants indicated that it was easy to remember putting the reference card near the food item before taking the image because they had learned the step during training. They acknowledged that they carried the reference card at the back of their phone covers and walked around with it all the time. One participant reported forgetting to take the fiducial marker when leaving the office sometimes but had found a solution after putting it in their wallet and hence carried it everywhere.

*“It was easy to remember putting the marker”.*

—P4

*“I was walking with my card behind my phone’s cover, so I was with it everywhere I went. So, wherever I go with my phone, I have my card as well. I didn’t face any problem”.*

—P15

*“I sometimes forgot It [fiducial card], and sometimes I reached far away, and when I realized that I had forgotten the card, I had to go back to take the card; that was the challenge I was facing. After observing that challenge, I decided to put the card into my wallet just like an ATM card so that I could go with it everywhere I go”.*

—P14

### 3.4. Potential for Applying the VISIDA App in Participants’ Work

#### 3.4.1. Acceptability

All participants liked how the VISIDA app collected dietary intake by capturing images and voice recordings of foods and drinks consumed. All participants expressed how much easier the VISIDA app would be at collecting the dietary intake of their clients compared to traditional methods. The participants were eager to start using the VISIDA app in their daily jobs, and those working at the hospital agreed that having the VISIDA app would improve the nutritional management of their clients.

*“I request that this program should be in Tanzania so that we can use it as professionals to manage problems associated with nutrition among our clients. It is better than other like 24-h recall or diet history where you cannot see any food that the patient eats”.*

—P2.

*“I would like to use it in my work because it is a good way compared to all other ways which are present”.*

—P4

*“To be honest, this is the best way because it doesn’t rely on information alone, but you get the image of foods that have been eaten, and it becomes easy to estimate the variety of foods eaten by a person and the amount that she has eaten”.*

—P14

#### 3.4.2. Training Others

All participants indicated that they could teach the use of the VISIDA app to others. However, they admitted that they would face challenges depending on the comprehension level of their trainees.

*“I can train someone without any problem, but I think to teach this app to a client who is just a layman can be a little bit difficult”.*

—P1

*“It will be easier for outpatient clients, you know according to patients’ sickness you can’t tell some of them to take photos of their meals”.*

—P12

*“VISIDA app is good, and I can train another person about it, but the challenge is on installing it”.*

—P21

#### 3.4.3. Participants’ Clients Using the App

Participants indicated that they had many clients with different socioeconomic characteristics, and they thought some of their clients would face some challenges in using the app. They had a few concerns for clients who had never used a smartphone and felt it could be difficult for them to master the app. Participants agreed that thorough training would help their clients understand and use the app.

*“If they [clients] will be trained it is possible and also for a person who has a smartphone after instructed her, I think she will not fail to use this one. Yes if you teach her about everything that she is supposed to do she will not fail to do so”.*

—P4

*“If the client comes from the rural areas and you want the person to use the app and does not have smartphone or s/he is not conversant with the smartphone, it will be a problem”.*

—P3

*“The majority of my clients will be able to use it. The challenge will be those people without smartphones, but for the majority of people with smartphones will be able [to use it]”.*

—P7

### 3.5. Perception on the VISIDA Web Platform for Analysing Dietary Intake Data

#### 3.5.1. General Views

Participants agreed that the VISIDA platform was a tool that could improve how they utilise their clients’ dietary intake data. Participants reported that they always estimate the intake of their clients by looking at which food group is missing, but they do not analyse nutrient intakes. They agreed that the VISIDA web platform could fill this gap. Participants were interested to learn more about how the VISIDA web platform works.

*“This platform is good, because it analyse everything from the beginning until you get complete information, that this person eats how much, so it helps to approximate meals that person has eaten, and you see what was eaten clearly”.*

—P4

*“It was easy to understand because everything is explained, so you know that when everything is explained, things become easier. How can I access this platform?”*

—P7

#### 3.5.2. Process for Coding (Identification and Quantification) of Collected Intake Data

Most participants were interested in the quantification process using VISIDA platform. However, they acknowledged that receiving training on using the platform and actually using the system versus only watching a video of it in action could place them in a better position to comment on this aspect of the platform.

*“Coding is good. I even saw a ruler as well when quantifying, adding measurement on food items. In general, I have seen that this is a good thing”.*

—P4

*“I think it is good because it will give you an estimation of the portion size of dietary intake of clients”.*

—P1

*“Maybe a person has taken photos of meals, and she didn’t say the amount of that food which she has eaten, so coding can approximate that this person has eaten a certain amount”.*

—P4

#### 3.5.3. Comparing Analysed Intake Data to Nutrient Intake Recommendations

Participants thought this was an essential part of the VISIDA platform. They agreed that because of this part, their work as nutritionists could be streamlined. Participants indicated that comparing intake against recommendation would help identify the gap they will need to fill when offering dietary advice to their clients.

*“This [comparing intake with recommendation] is very important section to direct me to whether my clients have taken the correct or incorrect amount of nutrients. This would help me to provide appropriate counselling”.*

—P2

*“This section [comparing intake with recommendations] is good because at the end of the day you get answers that this person gets the daily recommended dietary intake or not”.*

—P4

*“Because this part will tell you what the person ate, it will simplify things for nutritionists instead of [the need to] start making calculations”.*

—P15

### 3.6. Challenges Identified by the Participants

#### 3.6.1. Downloading the VISIDA App

Some participants had difficulty downloading the app due to an unstable internet connection. Where this occurred, participants consulted the first author (NS), who advised them to wait and download when the internet was stable, solving the problem.

*“In large part, the VISIDA app is good, as I have said in the beginning, just in installing it, I faced some problems”.*

—P19

#### 3.6.2. Taking Images with the VISIDA App

The majority of the participants acknowledged having faced some challenges in getting the right angle and balancing the cross (on-screen guidance) on the camera screen with the cross at the centre of the fiducial marker. In addition, they found it challenging to get the angle of the phone correct during the first day. Participants indicated that by the second day, it was easy to get the right angle to capture the image.

*“In setting the card and looking at those two dots together with mark ‘cross’, but doesn’t trouble after getting experience”.*

—P4

#### 3.6.3. Recording All Food and Drinks Consumed

Some participants indicated that it was a challenge to record in between meals (i.e., snacks). They stressed that they sometimes ate a snack on the bus while travelling back home or sometimes on the road, and therefore felt it was difficult to collect an image and voice recording.

*“So, I think to capture pictures of snacks eaten between meals was very tricky because sometimes I ate them while I was in a commuter bus”.*

—P11

*“It was a challenge to record snacks because it is easy to forget about little things”.*

—P3

*“The only challenge I got was on recording snacks I ate outside home, I mean the ones that I bought when I was passing in the streets especially in the evening”.*

—P7

#### 3.6.4. Recording in Different Places

The majority of the participants indicated that it was challenging to record their eating occasions outside their homes, as other people wondered what they were doing.

*“When I am at restaurant or cafeteria, there was a challenge of starting to take a picture, and that takes time, and everybody was wondering what is this person doing, I feel more comfortable at home compared to these other places”.*

—P2

*“Recording outside the home is a challenge in some areas, considering the setting of our work environment”.*

—P7

#### 3.6.5. Recording Recipes

Some participants indicated that it was challenging to record recipes, as they did not prepare food at home.

*“I wasn’t able to record cooking because most of the time I am at my workplace. I go back home late, and most time, I deal with children and my housemaid helps me cooking”.*

—P1

*“I like the VISIDA app, but unfortunately I didn’t get any chance of cooking”.*

—P16

### 3.7. Participants’ Recommendations

Participants gave the following recommendations, based on their experiences of using the VISIDA app to collect their dietary intakes.

- Enable the access of the VISIDA app from the app store like other smartphone apps. The following quote stresses:
  *“It should be simpler before using it, the way to download it, that it should be in the play store so that people can download it”.*
  —P1;
- The VISIDA app should be used in iOS smartphones and all versions of Android. These sentiments were expressed below:
  *“If possible VISIDA app should be used in all smartphone programs such as android and IOS”.*
  —P3
  *“My suggestion, if possible this [VISIDA] app should be used in all smartphones without looking android version above seven”.*
  —P4;
- The notifications should pop up with a sound. This was supported by the quote below:
  *“Notifications were popping up silently, I think it will be best if I could hear voice or vibrations when the notifications are popping up”.*
  —P3;
- Materials used to make a fiducial marker should be strong enough to resist harsh environments. The following opinion expresses this:
  *“And the materials which was used to make this card, it should be a certain materials either a certain plastic material which any person can use without being destroyed. The way I see it can be destroyed easily. If let say we give this to a mother, and she will take it, within a week it will be destroyed”.*
  —P7.

## 4. Discussion

The current study assessed the feasibility of using an image-based food record method by nutritionists in urban Tanzania. The study also explored the experiences and perceptions of the nutritionists on the VISIDA app and web platform for collecting and analysing intake data. It is the first study of its kind to be implemented in Tanzania. The image-based food record method evaluated was well accepted by the participants, with few challenges identified. Participants found the VISIDA app easy to use, and they showed interest in using the VISIDA system in their daily work.

Participants of this study reported usually conducting dietary assessments of their clients and agreed that it was essential to assess the dietary intake of their clients. The findings complement our previous study conducted among nurses working with pregnant women who indicated that dietary assessment was one of the services offered to their clients [18]. Participants in the current study assessed the dietary intakes of their clients mainly using 24-h recalls and/or FFQs. They mentioned disadvantages of using these traditional recall methods, such as their clients forgetting what they ate or potentially misreporting intake. It is well-documented that dietary information collected using 24-h recall and FFQ depends on the participants’ memory and requires the skills of a well-trained interviewer as a key step in minimizing recall bias [3].

The findings from this study demonstrate that it is feasible for trained nutritionists to use an image-based smartphone app to collect dietary intake in Tanzania. While the participants in the current study were employed nutrition professionals with regular use of and access to a smartphone, they agreed that most of their clients could use the VISIDA app. Participants indicated their preference in using the VISIDA app to collect dietary intake compared to traditional methods. This finding is consistent with previous studies which have used different dietary assessment methods with members of the general public. For example, in Australian adults with type 2 diabetes, there was a preference for using image-based food records over the ‘pen and paper’ traditional methods, such as a three-day weighed food record [16] and a three-day estimated food diary [19]. Additionally, a study in healthy adults in Canada reported that more participants (34.2%) preferred using the Keenoa app to collect their dietary intake compared to the traditional ‘pen and paper’ method, the 3-day food diary (9.6%) [20]. Although the nutritionists in our study felt that the VISIDA app could be used with their clients, this remains to be evaluated prior to making the app widely available in Tanzania.

Regarding usability, participants felt the VISIDA app was easy to use and felt they would be able to train others to use the app. Previous research with pregnant women in Australia found similar results, with participants reporting that it was easy to use the smartphone app to collect dietary intake [15]. Additionally, a study involving adolescents with type 1 diabetes in Finland found consistent results, with the majority (85%) finding it was easy to record food intake by mobile phone [21]. Participating nutritionists in the current study commented that it was easy to forget to record some eating occasions, particularly snacks and water, especially when consumed in places where it is hard to take photos, for example, in public transport. These findings are consistent with results from previous studies, which reported misreporting of snacks and food eaten at times other than the main meal when the image-based food record was used [16,22]. Furthermore, all participants were willing to use the image-based food record method again, which is similar to findings of a previous Australian study [15].

The participants reported recording the meals eaten away from home to be difficult as opposed to recording meals eaten at home. Some participants reported feeling more comfortable recording their eating occasions at home compared to restaurants and cafeterias because they felt that other customers were wondering why they took photos of foods. This is consistent with the results from the previous study that reported some participants were uncomfortable taking the food image in front of others [15]. Recording recipes was a challenge to most of the participants. The main reason is spending most of the time at work, and the presence of the house helps prepare food for the family.

Most participants reported that it was easy to carry around the fiducial marker and place it near food when recording eating occasions. It is likely that the VISIDA app’s on-screen guidance on placement of the marker assisted in this process. Similar observations were reported in a study conducted among adolescents in the United States of America [22]. Although one participant in the current study reported forgetting to carry a fiducial marker once, she found a way to take it around by placing it in her wallet, which overcame the challenge. Participants in a previous study, where no on-screen guidance was provided, who also used their own phones commented that it was difficult to remember to put the fiducial marker in the pictures [15]. In contrast, most of the participants in a different study, who were provided with a phone with a marker attached to the back of the phone, agreed that markers helped remind them how to use an image-based food record app [16]. Therefore, ensuring that the process of using a fiducial marker is as simple as possible is a key in the future use of the VISIDA smartphone app.

This study demonstrated that remotely training nutritionists to use the VISIDA app was feasible. However, there were some unforeseen issues with some participants’ phones, along with challenges in accessing a cloud storage platform (designed for the study and not part of the VISIDA system) to upload the exported VISIDA app data. The key recommendation given by participants for improving the app was of including an alarm for notifications. Although the VISIDA app does already have an inbuilt notification system, participants can control the volumes of the ringtone, media, notifications and system independently on the Android OS. Therefore, ensuring that the volume for notifications is set to an appropriate level is essential and will be expanded in the revised training materials for the VISIDA smartphone app.

A strength of the current study is that it is the first study to determine the feasibility of using image-based food records in Tanzania. A limitation of this study is that findings cannot be generalised to Tanzania’s general population. The current study recruited highly educated nutrition professionals and employed participants with experience in conducting dietary assessments and with knowledge and experience in using a smartphone. Another limitation is that the participating nutritionists worked in urban settings; hence, evaluating the feasibility for nutritionists in rural settings to be trained remotely would need to be established. Additionally, nutritionists who were not FONATA members were not invited to participate, and therefore, sampling bias could occur. However, the recruited nutritionists covered varied practice areas such as research, clinical, public health, and education. Thus, the findings are likely to be representative of the nutritionists working in these sectors.

## 5. Conclusions

The VISIDA app for collecting image-based food records functioned well in a Tanzanian context and was well-accepted by the nutritionists who participated in the current study. Participants found the VISIDA app was easy to use in the collection of their dietary intake data and demonstrated interest in using the method in their daily roles. Participants recommended adding sound on notifications, an Apple iOS version of the VISIDA app in addition to the Android version, enabling installation directly from the app store, and improving materials for making the fiducial markers. Image-based food records could be used in urban Tanzania to enhance the assessment of dietary intake by nutritionists. Further research is needed to understand the usability and acceptability of the image-based food record method by the general population in Tanzania.

## Figures and Tables

**Table 1:** Data collection framework for the nutritionists’ study.

Week	Activities Completed and Data Collected
Week 1	− Participants received a link to the VISIDA app to download to their phones and an instruction video on how to install the VISIDA app. − Participants received virtual training individually for an hour on how to use the VISIDA app over a video call using the Zoom platform (https://zoom.us/ accessed on 30 September 2021). This included how to get the right camera angle by aligning the cross in the on-screen guidance on the camera screen with the cross at the centre of the fiducial marker. − Participants collected dietary intake and food preparation information using the VISIDA app for three days. Participants finalised their eating by reporting what happened to the recorded eating occasion, and they took a picture of leftovers, if any. At the end of the day, participants reviewed their day and were able to voice record any forgotten foods and drinks.
Week 2	− Participants were asked to export the collected data and upload it to a link provided by the research team. − Participants filled a questionnaire regarding their experience using the smartphone app to collect dietary information. − Participants received a VISIDA web platform video link and watched the video that demonstrated how the VISIDA web platform analyses intake data. − Participants were interviewed via ZOOM regarding their experience in using the VISIDA app and their perceptions of the analysis process using the VISIDA web platform.

**Table 2:** Participants’ characteristics.

Characteristic	Frequency	Percent
**Profession (Role)**		
Nutritionist (clinical)	11	61.1
Nutritionist (research)	4	22.2
Nutritionist (public health)	2	11.1
Nutritionist (teaching)	1	5.6
**Working experience (years)**		
0–1	5	27.8
2–3	3	16.7
4 and above	10	55.6
**Education level**		
Bachelor	15	83.3
Postgraduate	3	16.7
**Gender**		
Male	3	16.7
Female	15	83.3
**Place of work**		
Government hospital	11	61.1
Government institution	4	22.2
Private secondary school	1	5.6
Non-governmental organisation	2	11.1
**Experience in using smartphone**		
A lot of knowledge	13	72.2
Some knowledge	5	27.8
** Experience in working with		
Babies (0–12 month)	8	44.4
Children (13–59 month)	11	61.1
Children (5–9 years)	4	22.2
Youth (10–19 years)	4	22.2
Pregnant women	17	94.4
Lactating mothers	8	44.4
Women ≥ 20 (not pregnant/lactating)	9	50
Men ≥ 20	10	55.6

** Participants could select multiple options for this question.

**Table 3:** Perceptions of the participants on the usability and acceptability of the VISIDA app.

Variable	Strongly Disagree	Disagree	Neutral	Agree	Strongly Agree
It was easy to take photos of food/drink	0 (0)	1(5.6)	3 (16.7)	8 (44.4)	6 (33.3)
Easy to make voice records of food/drink	0 (0)	0 (0)	0 (0)	4 (22.2)	14 (77.8)
Easy to remember to record food/drink before eating	0 (0)	4 (22.2)	7 (38.9)	6 (33.3)	1 (5.6)
Easy to remember to record leftovers	1 (5.6)	2 (11.1)	2 (11.1)	10(55.6)	3 (16.7)
Easy to remember to include fiducial markers in food/drink	1 (5.6)	1 (5.6)	2 (11.1)	6 (33.3)	8 (44.4)
**Recording food/drink intake:**					
Disrupted daily activities	8 (44.4)	6 (33.3)	3 (16.7)	0 (0)	1 (5.6)
Disrupted meal times	10 (55.6)	5 (27.8)	2 (11.1)	0 (0)	1 (5.6)
Changed the types of food/drinks consumed	10 (55.6)	3 (16.7)	2 (11.1)	2 (11.1)	1 (5.6)
Changed the amount of food/drinks consumed	12 (66.7)	2 (11.1)	2 (11.1)	1 (5.6)	1 (5.6)
Changed the frequency of consuming food/drink	11 (61.1)	3 (16.7)	2 (11.1)	1 (5.6)	1 (5.6)

## Data Availability

The data presented in this study are available on request from the corresponding author.
